# ANCA vasculitis presenting with acute interstitial nephritis without glomerular involvement 

**DOI:** 10.5414/CNCS109805

**Published:** 2019-07-19

**Authors:** Callie Plafkin, Weixiong Zhong, Tripti Singh

**Affiliations:** University of Wisconsin School of Medicine and Public Health, Department of Medicine, Madison, WI, USA

**Keywords:** ANCA-associated vasculitis, interstitial nephritis, MPO-ANCA

## Abstract

ANCA-associated vasculitis (AAV) with renal involvement typically causes pauci-immune glomerulonephritis. We present a case of acute interstitial nephritis (AIN) as the sole renal lesion without glomerulonephritis with myeloperoxidase (MPO) AAV. A 45-year-old female with history of Crohn’s disease, gastroesophageal reflux disease (GERD) with Barrett’s esophagus, pulmonary embolism, and polyarthralgias was evaluated in nephrology clinic in 2018. AIN without glomerulonephritis was first diagnosed in 2011 on renal biopsy. p-ANCA was positive with MPO titer of > 100 U/mL. Serum creatinine improved from 2.1 to 0.9 mg/dL with prednisone and azathioprine. Repeat biopsy in 2013 for worsening renal function showed AIN without glomerular involvement. Serum creatinine improved from 1.9 to 1.2 mg/dL with prednisone and cyclosporine. Crohn’s disease was diagnosed in 2014. AIN was attributed to Crohn’s, and cyclosporine was stopped in 2016. Adalimumab was started in 2016, without improvement in renal function or urine sediment. Attempt was made to switch proton pump inhibitor (PPI) to H2-blocker, but the latter was not tolerated. Repeat biopsy in 2/2018 showed AIN with severe fibrosis and tubular atrophy and glomerulosclerosis but no active glomerular disease. MPO titers remained high at 132 U/mL. Mycophenolic acid and prednisone were started without response, followed by rituximab for AAV-associated AIN. Serum creatinine worsened to 6.0 mg/dL in 9/2018, with plan to start peritoneal dialysis. AAV may present with isolated AIN without glomerular involvement. The rarity of this presentation may contribute to delay in appropriate management. Alternative explanations for AIN, such as Crohn’s disease or PPI use should be considered with caution in the setting of high-titer ANCA positivity.

## Introduction 

Renal involvement is common in myeloperoxidase (MPO) ANCA-associated vasculitis (AAV), which typically manifests as pauci-immune crescentic glomerulonephritis (GN). Tubulointerstitial lesions can be seen in AAV-associated GN but are thought to be secondary to rupture of Bowman’s capsule and crescent formation rather than representing an independent process [1]. There are no cases of proteinase 3 (PR3) AAV, and only a few reports of cases of MPO-ANCA-associated vasculitis, presenting as isolated interstitial nephritis without glomerular involvement [2, 3, 4]. We present such a case. 

## Case report 

**Figure 1. Figure1:**
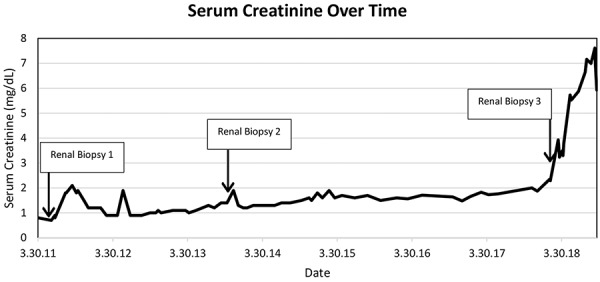
Serum creatinine trend over time.

**Figure 2.**
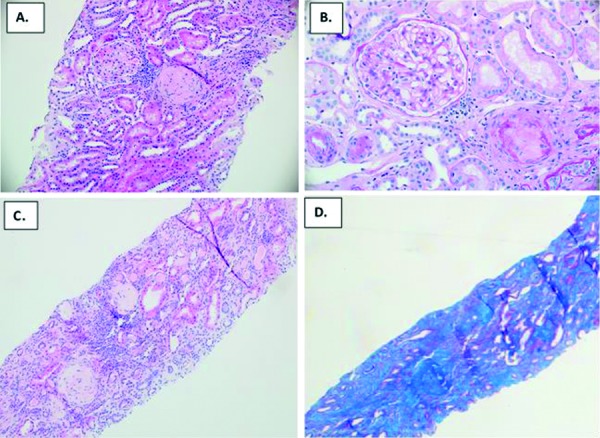
A: Hematoxylin and eosin (H & E) stain of initial (6/2011) renal biopsy, × 20 magnification, demonstrating interstitial inflammatory infiltrate with normal glomerulus. B: H & E stain of initial renal biopsy (6/2011), × 40 magnification, depicting normal glomerulus and no glomerular hypercellularity, fibrinoid necrosis or crescent. C: H & E stain of third renal biopsy (2/2018), showing persistent interstitial inflammatory cell infiltrate and glomerulosclerosis. D: Trichrome stain of third (2/2018) renal biopsy, highlighting diffuse, extensive interstitial fibrosis and global glomerulosclerosis (blue).

A 45-year-old female with a history of Crohn’s disease, recurrent sinusitis, presumed pulmonary embolism, gastroesophageal reflux disease (GERD) with Barrett’s esophagus, endometriosis, and polyarticular arthralgias was evaluated in our nephrology clinic in 2018[Fig Figure1]. Renal disease evaluation began in March of 2011, when she was noted to have microscopic hematuria and significant proteinuria, with urine protein-to-creatinine ratio of 2.1 mg/g. At that time, renal function was normal, with a baseline serum creatinine of 0.7 – 0.9 mg/dL in January through June 2011. She underwent renal biopsy in June 2011, which showed acute interstitial nephritis, mild interstitial fibrosis and tubular atrophy, and mild focal hyaline arteriosclerosis. There was no evidence of active glomerulonephritis (Figure 2A, B). Serologic testing was notable for a positive p-ANCA, with MPO titer of > 100 units/mL. Also in June of 2011, she noted arthralgias affecting the hands and feet, with ~ 1 hour of daily morning stiffness. 

Additionally, she was treated in June for a presumed pneumonia, based on symptoms of cough and pleuritic chest pain, with findings of a left lower lobe infiltrate noted on chest X-ray and non-contrast-enhanced chest CT. This was later thought to represent pulmonary embolism with pulmonary infarction based on a pulmonologist’s interpretation of her imaging and clinical presentation. Notably, she did not undergo dedicated CT angiography due to a contrast allergy. Her estrogen-containing oral contraceptive, prescribed that year for management of dysfunctional uterine bleeding, was discontinued, and she received anticoagulation with low molecular weight heparin followed by warfarin for presumed pulmonary embolism. 

In August through September of 2011, proteinuria increased to 3.3 g/day and serum creatinine increased to 2.1 mg/dL. She was started on prednisone and azathioprine, with improvement in serum creatinine to 0.9 mg/dL by February 2012. Prednisone was slowly tapered. She noted that fatigue and arthralgias improved with prednisone but recurred upon tapering. 

She was referred to Rheumatology in early 2012 for further evaluation of arthralgias, proteinuria, and strongly positive anti-MPO antibody. Although she had no evidence of active synovitis on exam, inflammatory polyarticular arthritis was suspected. She also described recurrent sinusitis which had consistently improved in the past with antibiotics; she had no sinus complaints at the time of evaluation. She denied any rashes, dyspnea, hemoptysis, or focal neurologic complaints. There was no evidence of rash, pulmonary abnormalities, or neurologic deficits on exam at the time of evaluation. Due to prior rise in serum creatinine and polyarticular arthralgias with suspicion for inflammatory polyarthritis, and strongly positive anti-MPO antibody, her rheumatologist expressed concern for the possibility of microscopic polyangiitis or granulomatous polyangiitis, urging consideration of more aggressive immunosuppression if renal function continued to worsen. However, due to lack of characteristic AAV renal findings (glomerulonephritis), no changes were made to her immunosuppressive therapy at that time. 

In July to November 2013, renal function again worsened to a peak serum creatinine of 1.9 mg/dL. Repeat biopsy in October 2013 again showed acute interstitial nephritis without vasculitic glomerular involvement, as well as secondary glomerulosclerosis and mild interstitial fibrosis and tubular atrophy. Prednisone and cyclosporine were started, with an improvement in serum creatinine to 1.2 – 1.4 mg/dL. Proton pump inhibitor (PPI) discontinuation was also attempted, but poorly tolerated due to worsening of reflux symptoms on ranitidine; omeprazole was resumed in late April 2014. 

In 2014, she was hospitalized with abdominal pain and diagnosed with Crohn’s disease on colonoscopy, with ileal and left colonic involvement, and was started on adalimumab therapy. Acute interstitial nephritis was attributed to Crohn’s disease and cyclosporine was stopped in 2016. Although her gastrointestinal symptoms improved on adalimumab, renal function gradually worsened, with serum creatinine increasing to 1.5 – 1.7 mg/dL in 2015 – 2016, 1.7 – 2.0 mg/dL in 2017, then 2.3 mg/dL in February 2018, at which time a third renal biopsy was performed. This again demonstrated active interstitial nephritis without glomerulonephritis, now with severe interstitial fibrosis and tubular atrophy involving over 80% of the cortical parenchyma and global glomerulosclerosis (Figure 2C, D). MPO titers remained high at 132 U/mL. 

She was started on mycophenolic acid and prednisone without significant response, followed by rituximab induction therapy for a diagnosis of AAV-associated active interstitial nephritis. Expectation was low for robust renal recovery, given the extent of renal fibrosis and irreversible damage already present by the time of induction immunosuppressive therapy. Serum creatinine continued to worsen to 5.9 – 7.6 mg/dL, and glomerular filtration rate is now consistently less than 10 mL/min/1.73m^2^ (Figure 1). Preparations are being made to start peritoneal dialysis. 

## Discussion and conclusion 

The characteristic renal lesion observed in AAV is acute necrotizing crescentic GN. Though mononuclear tubulointerstitial infiltrates are also frequently observed in AAV, they are usually seen in conjunction with GN [1]. Our patient had only isolated interstitial nephritis without any evidence of GN on three repeat renal biopsies. High-titer MPO-ANCA persisted throughout her disease course. In retrospect, the patient’s report of recurrent sinusitis, polyarticular arthralgias, and pulmonary infiltrate treated as pneumonia during the year of presentation may have represented other systemic manifestations of MPO-AAV. Literature strongly supports the pathogenic role of MPO-ANCAs in the development of MPO-AAV, and there is evidence for correlation between MPO-ANCA titers and disease activity [5, 6, 7]. It is important to recognize this atypical presentation of AAV, and to consider the diagnosis of AAV in patients with progressive renal disease and persistent high-titer ANCA, even in the absence of GN on kidney biopsy. 

Antineutrophilic cytoplasmic antibodies are not only of diagnostic value in AAV; they have also been shown to play a pathogenic role in disease development. A key case report by Bansal and Tobin [8] from 2004 illustrated the pathogenic role of MPO-ANCA in a neonate who developed pulmonary hemorrhage and glomerulonephritis following transplacental transfer of maternal anti-MPO antibodies. Pathogenic mechanisms of anti-MPO antibodies in vasculitis have been elucidated in vitro, initially by Falk et al. [9] who described the process of ANCA-induced neutrophil degranulation in 1990. When primed by pro-inflammatory cytokines, neutrophils express MPO molecules on the cell surface. MPO-ANCAs then bind these MPO antigens and trigger neutrophil degranulation, leading to release of reactive oxygen species and lytic enzymes, and consequent endothelial cell damage [9, 10]. Normally, the physiologic response to infection or injury involves leukocyte activation and diapedesis across the post-capillary venule wall, without full leukocyte activation until after it migrates away from the vessel wall. In MPO-AAV, leukocytes become fully activated by ANCA within the vessel (mural activation), resulting in endothelial damage and small-vessel vasculitis [11]. MPO-ANCA has also been shown in vivo in the mouse model to induce vasculitis and crescentic GN following injection of anti-MPO IgG into wild-type mice [10, 12, 13]. Multiple studies have found that serum titers of MPO-ANCA correlate with AAV disease activity and risk of relapse [5, 6, 7]. 

It has been postulated that AAV renal lesions may exist along a spectrum, including both interstitial nephritis and crescentic GN, with tubulointerstitial nephritis (TIN) representing a more benign presentation along this spectrum [14]. In 2006, Wen and Chen [14] described a case of acute renal failure in a patient with MPO-ANCA in whom initial biopsy showed TIN, with subsequent transformation into crescentic glomerulonephritis on repeat biopsy. It remained unclear whether this transformation represented natural history of the disease, or a disease variant. Interestingly, our patient’s course differs in that renal dysfunction progressed to stage 5 chronic kidney disease despite repeat renal biopsies showing only interstitial nephritis without ever demonstrating GN histopathology. However, the repeat biopsy showed worsening glomerulosclerosis which was the cause of nephrotic range proteinuria seen in our patient at the time of the third biopsy. The glomerulosclerosis could have been due to undiagnosed crescentic glomerulonephritis as a result of sampling error in the first two biopsies or as a result of extensive interstitial fibrosis and tubular atrophy due to prior untreated active TIN. Isolated acute TIN has been described previously in several other case reports, and only in association with MPO-ANCA [2, 3, 4]. 

Initially, our patient had multiple plausible alternative explanations for TIN, including Crohn’s disease, treatment with adalimumab, and PPI therapy. Despite some associations of adalimumab therapy with interstitial nephritis, this was determined an unlikely culprit of the patient’s disease given the time course, as TIN preceded initiation of adalimumab. Unfortunately, discontinuation of PPI therapy was not tolerated. Though interstitial nephritis has been observed in Crohn’s disease [15], renal disease failed to improve with Crohn’s therapy in our patient. Reliance on these alternative explanations for TIN contributed to diagnostic delay, with progression of our patient’s renal failure. In patients with TIN and MPO-ANCA, strong consideration should be given to the possibility of AAV, even in the absence of glomerular lesions. Alternative explanations for interstitial nephritis should be considered with caution in the setting of high-titer ANCA positivity. 

## Funding 

There were no specific funding sources relevant to this manuscript. 

## Conflict of interest 

The authors have no conflict of interest to declare.
